# Safety of a formulation containing chitosan microparticles with chamomile: blind controlled clinical trial*

**DOI:** 10.1590/1518-8345.2648.3075

**Published:** 2018-11-29

**Authors:** Danielle Cristina Garbuio, Cristina Mara Zamarioli, Maísa Oliveira de Melo, Patrícia Maria Berardo Gonçalves Maia Campos, Emília Campos de Carvalho, Luis Alexandre Pedro de Freitas

**Affiliations:** 1 Universidade Anhanguera, Valinhos, SP, Brazil.; 2 Universidade de São Paulo, Escola de Enfermagem de Ribeirão Preto, PAHO/WHO Collaborating Centre for Nursing Research Development, Ribeirão Preto, SP, Brazil.; 3 Universidade de São Paulo, Faculdade de Ciências Farmacêuticas de Ribeirão Preto, Ribeirão Preto, SP, Brazil.

**Keywords:** Matricaria, Dermatologic Agents, Prevention and Control, Nursing, Clinical Trial, Medicinal Plants

## Abstract

**Objective:**

to evaluate the safety of a topical formulation containing chamomile microparticles coated with chitosan in the skin of healthy participants.

**Method:**

phase I blind, controlled, non-randomized, single-dose clinical trial with control for skin, base formulation, and formulation with microparticles. The variables analyzed were irritation and hydration by the Wilcoxon and Kruskall-Wallis tests.

**Results:**

the study started with 35 participants with a mean age of 26.3 years. Of these, 30 (85.71%) were female, 29 (82.90%) were white skinned and 32 (91.40%) had no previous pathologies. One participant was removed from the study reporting erythema at the site of application, and four other participants for not attending the last evaluation. In the 30 participants who completed the study, the tested formulation did not cause erythema, peeling, burning, pruritus or pain; there was an improvement in cutaneous hydration in the site of application of the formulation with microparticles. In the evaluation of the barrier function, there was an increase in transepidermal water loss in all sites.

**Conclusion:**

the formulation with chamomile microparticles is safe for topical use, not causing irritation and improving skin hydration over four weeks of use. Its effects on barrier function need further investigation. N^o^. RBR-3h78kz in the Brazilian Registry of Clinical Trials (ReBEC).

## Introduction

The skin, as the interface of the human body with the external environment, carries part of our identity: it provides information about our age, genetics, health status, lifestyle and even our emotional state^1^. The skin has different roles, among them, the function of barrier, thermoregulation, vitamin D synthesis and also protection of the body against harmful agents^2^ ^-^ ^3^.

Currently, there is a growing interest on skin care products and their protective and healing properties, especially those with botanical extracts^4^. Among the latter, *Chamomilla recutita* (L.) rauschert (chamomile) is a popular plant^5^ which has its use as a phytotherapic released by the National Agency of Sanitary Surveillance (ANVISA)^6^. Chamomile has flavonoids, among which apigenin and apigenin-7-glycoside are the most abundant^7^. In different studies, these substances have proved to have antimicrobial^8^, analgesic^9^, anti-inflammatory^6^ ^,^ ^8^ ^,^ ^10^ ^-^ ^11^, cicatrizant^12^ ^-^ ^13^, antitumor^14^ ^-^ ^15^ and immunomodulator^16^ potential. Moreover, the antioxidant blend present in chamomile extract is effective in reducing free radicals and brings potential benefits when used in skin formulations by reducing water loss, improving hydration, and aiding the maintenance of the barrier function^4^.

The risks associated with the use of this plant are small and are related to the reduction of platelet aggregation^17^ and anaphylactic reactions to people sensitive to its components^18^. However, a recent study that evaluated allergic reactions to herbal compounds over the last 27 years found no reports related to *Chamomilla recutita* (L.) rauschert^19^.

Despite the potential of apigenin and apigenin-7-glycoside, they have low stability^15^ ^,^ ^20^ ^-^ ^22^. An alternative to improve this issue is the use of controlled release systems. The pharmaceutical sector, in line with technological developments, has gradually improved the processes of obtaining different products and invested in the forms of producing and applying them. To this end, microencapsulation is a widely used technology aimed at optimizing industrial processes as well as increasing the bioavailability and stability of the formulations. This process can be accomplished by different methods, using various coatings. In this study, the selected coating was chitosan and the method of production was spray drying^21^.

Chitosan is a biocompatible, biodegradable, low-toxicity hydrophilic polysaccharide with mucoadhesive and film-forming properties^23^. Studies point to its potential in the treatment of skin lesions, with positive results in cicatrization and inflammatory response^24^ ^-^ ^28^. This polymer was also used to coat microparticles containing endothelial and epidermal growth factors, with positive results in improving the cicatrization process^29^, and also as a coating of microparticles capable of capturing and expanding specific cells in order to accelerate the anti-inflammatory and cicatrization processes^30^.

Despite all the potentialities of chitosan-coated microparticles and the therapeutic properties of chamomile, no studies with these compounds were identified in the literature. These microparticles are incorporated in lanolin-based formulation, a safe substance for topical applications that incorporates various bioactive agents^31^.

In view of the above, a study was carried out to evaluate the safety of the topical formulation containing chitosan-coated *Chamomilla recutita* (L.) Rauschert microparticles for application on the skin of healthy volunteers evaluating the following variables: erythema, variation in the amount of melanin, desquamation, burning, pruritus, pain and alterations in cutaneous hydration. The hypothesis was that the use of this formulation would be safe for cutaneous application over four weeks of use.

## Method

Blind, controlled, non-randomized, single-dose Phase I clinical trial in which a low dose with biological activity of the active ingredient was administered^32^.

Extraction and microencapsulation methodologies developed and validated in a previous study were applied for the development of the microparticles used in this study^21^. Quality tests of the plant acquired according to the guidelines of the Brazilian Pharmacopoeia^34^ were carried out in another study developed by the main author, as well as preliminary permeation and stability tests of the formulation in an *ex vivo* model.

The formulations were prepared at the Laboratory of Industrial Pharmaceutical Development of the School of Pharmaceutical Sciences of Ribeirão Preto, University of São Paulo (LADIFARP/FCFRP/USP). The formulation with Chamomile (F1) had 99.8% of Lanolin and 0.2% of microparticles, and Formulation without Chamomile (F2) had 100% of Lanolin. The dose of chamomile in the microparticles and consequently in the formulation was selected based on the guidelines established by ANVISA^6^ for the amount of apigenin-7-glycoside.

The criteria for inclusion of participants were: age (18 years and over); healthy skin in the site of application of the product (forearms); absence of history of hypersensitivity to fish, seafood or any component of the formulation (chamomile, chitosan or lanolin); non-use of heparin, oral anticoagulants, and antiplatelet agents. The exclusion criteria were: injury at the sites of application, express intention to stop participation, and non-application of the product for more than four consecutive days.

National^35^ and international^32^ recommendations about studies aiming at the initial evaluations of tolerance and safety in healthy humans were considered; the participation of 20 to 100 individuals is recommended.

The study was carried out in partnership with the Nucleus of Advanced Studies in Cosmetic Technology at the School of Pharmaceutical Sciences of Ribeirão Preto, University of São Paulo (NEATEC/FCFRP/USP), whose laboratory is equipped for skin analysis. Participants were recruited through an invitation made personally by the principal investigator in the university during the period from August to September 2015.

**Figure 1:**
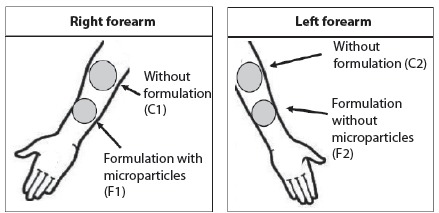
Scheme of sites of application of the product, control sites and site of evaluation of the skin

The four evaluation sites of each participant were: two control sites of skin without application of any product, one in each forearm (C1 and C2); one control site of application of the formulation (F2); and one experimental site (F1). Thus, each participant presented four evaluation sites, two with application of formulations and two without application of formulations (Figure 1).

For the application of the formulations, the procedures were standardized as follows: the application site should be free of any spot, lesions, irritations or abrasions, as set out in the inclusion criteria; the amount of formulation was pre-determined at the tip of a spatula and applied separately; formulation 1 (F1) was applied on the anterior side of the right forearm and formulation (F2) on the anterior side of the left forearm; in the proximal portion of both, no product was applied to allow assessment of skin conditions (right negative control - C1; left negative control - C2). The dose was applied once a day, daily, always at the same time, and the participants were instructed not to use any other product at the application and evaluation sites during the 28 days of study.

Before each evaluation, all participants remained in a room with conditioned airat 24ºC (± 2ºC), with relative air humidity (RH) around 50% (± 4%), for 15 minutes. The skin area evaluated was demarcated with a ruler, to help performing all evaluations in the same sites. On the first day of treatment (D0), an initial evaluation of the skin was made and the participants were instructed about the procedures of application of the formulations and the standardization of the sites. Each participant received two pots containing information on expiration date and application, one for the left forearm and one for the right forearm. All participants used both formulations.

After 24 hours (D1) of the first application, the participants were evaluated for tolerance and onset of adverse reactions. If no complaints or signs of irritation were reported, the treatment was continued until the day D28, when the participants were re-evaluated.

The outcomes analyzed were skin irritation and hydration, evaluated by means of quantitative measurement instruments, clinical evaluation and subjective evaluation. Quantitative determinations of erythema and melanin were performed with Mexameter^®^ MX18 (Courage and Khazaka Electronics Ltd, Koeln, Germany) and local skin pH was measured with a Skin-pH-Meter pH 900^®^ (Courage and Khazaka Electronics Ltd, Koeln, Germany). The visual clinical evaluation of macroscopic desquamation and the subjective evaluation were performed by inquiring the participants about the sensation of heat, burning, pruritus and pain. Skin hydration was evaluated by: the water content retained in the stratum corneum assessed with a Corneometer^®^ CM 825 (Courage & Khazaka, Koeln, Germany); the transepidermal water loss assessed with a Tewameter^®^ TM 210 (Courage and Khazaka Electronics Ltd, Koeln, Germany); clinical parameters of opacity and roughness; and a subjective evaluation of the participants regarding the sensation of hydration.

Quantitative measurements were performed by a pharmacist with experience in these analyses. The clinical evaluation was done by a nurse and the subjective evaluation by the participants themselves. All the results were annotated in a data collection instrument previously submitted to three judges for the evaluation of content and face validity.

The nurse and pharmacist who conducted the evaluations, as well as the participants, did not know which formulation contained the chitosan microparticles with chamomile. Furthermore, given the typical odor and the consistency of Lanolin^®^, the control formulation (F2) presented the same appearance as the one in the test (F1).

A descriptive analysis of each variable was performed and their distribution was verified by the Kolmogorov-Smirnov test; the appropriate statistical test was chosen only after this test. The analyses were run in the SAS^®^ software; a significance level (α) of 5% and power of 80% were adopted. The Wilcoxon test was chosen to evaluate the situation before and after the experiment in each application site. The Kruskal-Wallis test was used to compare the application sites of the formulations (F1 and F2) and controls (C1 and C2) at each moment.

The study was approved by the Research Ethics Committee of the College of Nursing of Ribeirão Preto (EERP/USP) under number 1,177,590/2015.

## Results

**Figure 2:**
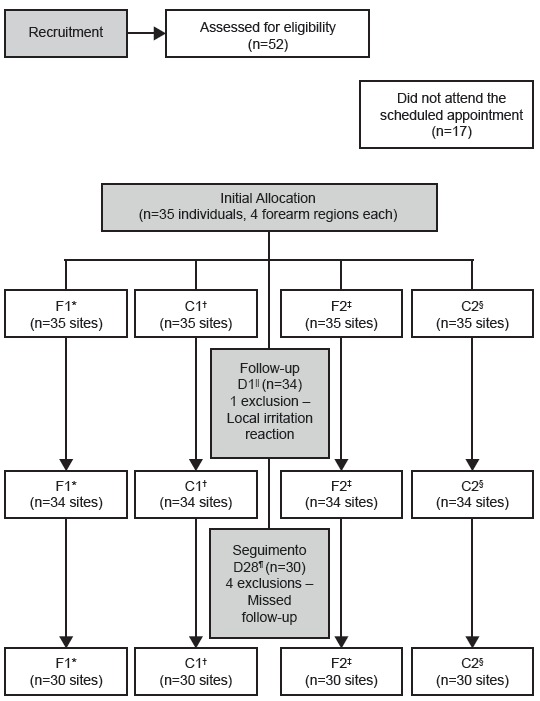
Flowchart of study participants according to CONSORT recommendations

A total of 52 participants were evaluated for eligibility and recruited by the principal investigator. Of these, 17 did not attend the appointment; thus, 35 participants were allocated to the study. The study was completed after four weeks, according to the initial schedule plan. After initiation, one participant was removed from the study because of local irritation. He reported small red dots on the right forearm three hours after applying the formulation (F1), with spontaneous regression about 30 minutes later. At the time of the evaluation of the D1, the site was unchanged and the participant reported no other complaints. For safety, this participant was instructed not to use the formulations and was removed from the study. Another four participants did not attend the final evaluation (D28), not concluding the proposed schedule. However, contact with these participants was maintained up to 21 days after application, when the date of the final evaluation was confirmed. We emphasize that until that date, none of these four participants had manifested any adverse reaction to the treatment. The flow of participants is shown in Figure 2.

* F1: Formulation with microparticles; ^†^C1: Without formulation; ^‡^F2: Formulation without microparticles; ^§^C2: Without formulation; ^||^D1: First day; ^¶^D28: Last day.

The mean age of the participants was 26.30 years (standard deviation 7.80, minimum 19, maximum 59), average body mass index (BMI) of 23.20 (standard deviation 4.42, minimum 16.1 , maximum 32.1). Other variables analyzed in the initial characterization of the participants are presented in Table 1.

**Table 1:** Sociodemographic and clinical characterization of participants at baseline (n = 35). Ribeirão Preto, SP, Brazil, 2015

Variables	n (%)
Sex	
Female	30 (85.7)
Male	5 (14.3)
Race	
White	29 (82.9)
Black	1 (2.9)
Brown	3 (8.6)
Asian	2 (5.7)
Personal history	
None	32 (91.4)
DM *	1 (2.9)
Other	2 (5.7)
Alcohol consumption	
No	5 (14.3)
Yes	0 (0.0)
Ex-alcoholic	1 (2.9)
Sporadic	29 (82.9)
Smoking	
No	32 (91.4)
Yes	0 (0.0)
Ex-smoker	3 (8.6)
Sporadic	0 (0.0)

*DM: Diabetes Melitus

Firstly, an evaluation was carried out to identify possible differences in erythema, melanin, pH, hydration and transepidermal water loss between the four sites assessed at the beginning (D0) and at the end of the treatment (D28). These data are presented in Table 2.

**Table 2:** Comparison of the means of erythema, melanin, pH, hydration and transepidermal water loss between the sites evaluated at the beginning (D0) and at the end of the treatment (D28) (n = 30). Ribeirão Preto, SP, Brazil, 2015

Variables	Beginning		End	
	Mean	p-value*	Mean	p-value*
Erythema				
F1^†^	598.9	0.6464	599.1	0.8796
F2 ^‡^	602.0		602.5	
C1^§^	601.1		603.8	
C2^||^	597.8		601.7	
Melanin				
F1^†^	483.1	0.0037^¶^	482.4	0.0053^¶^
F2 ^‡^	486.4		487.3	
C1^§^	496.0		498.4	
C2^||^	492.9		496.3	
pH				
F1^†^	4.1	0.0819	4.6	0.0019^¶^
F2 ^‡^	3.8		3.9	
C1^§^	3.7		4.4	
C2^||^	3.3		3.7	
Water content				
F1^†^	36.8	0.4837	40.3	0.1348
F2 ^‡^	37.3		40.3	
C1^§^	38.8		43.1	
C2^||^	36.0		38.6	
TEWL**				
F1^†^	6.2	0.0006^¶^	9.1	0.0029^¶^
F2 ^‡^	6.6		9.4	
C1^§^	4.5		7.4	
C2^||^	5.9		8.7	

*Kruskal-Wallis test; †F1: Formulation with icroparticles; ‡F2: Formulation without microparticles; §C1: Without formulation; ||C2: Without formulation; ¶p-value < 0.05; **TEWL: Transepidermal water loss

**Figure 3:**
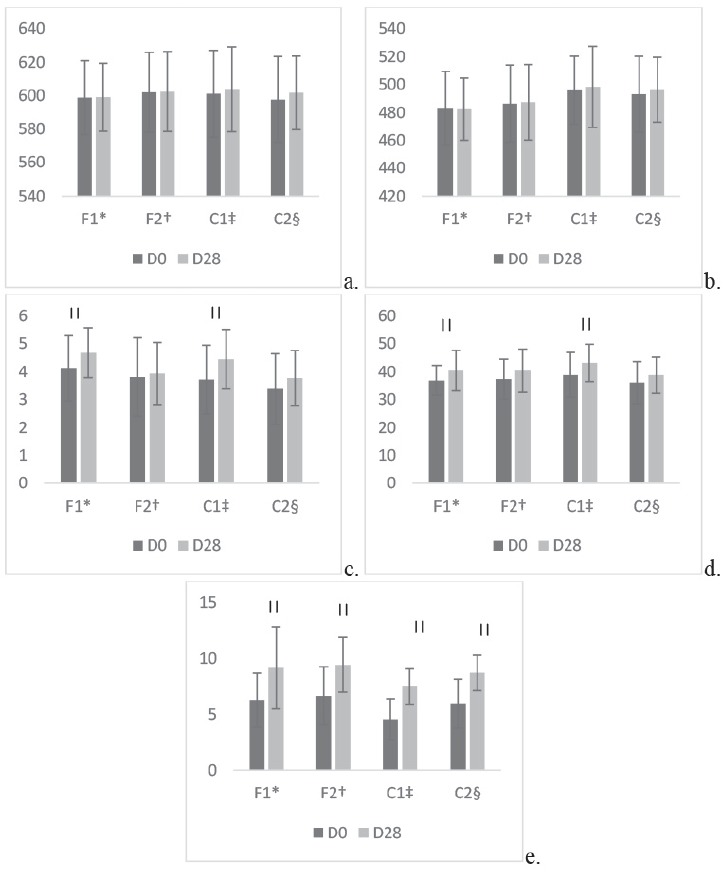
Distribution of the mean values of erythema (a), melanin (b), pH (c), hydration (d) and TEWL (e) at the initial (D0) and final (D28) days in each evaluated site

Another evaluation was performed comparing each site before (D0) and after treatment (D28) in order to identify whether the use of the formulation significantly altered the mentioned parameters. Thus, no changes were observed in the clinical evaluation of the parameters of desquamation and edema after the first day of application or at the end of the four weeks. There was no difference between the initial and final mean values for erythema and melanin variables at any of the application sites, which confirms the expected results. This same analysis for the pH averages showed a difference between the initial and final values at the application site of the formulation with microparticles (p = 0.0492) and at the right control site (p = 0.0303) (Figure 3).

*F1: Formulation with microparticles; ^†^F2: Formulation without microparticles; ^‡^C1: Without formulation; ^§^C2: Without formulation. ^||^ p-values less than 0.05 (Wilcoxon Test).

There was an increase in the average hydration coefficient at the site of application of the formulation with microparticles (F1) (p = 0.0483) and in the right control (C1) (p = 0.0413). In relation to the skin barrier function, there was an increase in the average of transepidermal water loss (TEWL) in the four evaluated sites (F1 - p = 0.0003; F2 - p = 0.0004; C1 - p < 0.0001; C2 - p <0.0001), indicating a decrease in the barrier function (Figure 3).

The clinical evaluation of irritation did not find desquamation in any participant in the evaluated period. As for the subjective parameters of irritation (pain, burning, pruritus and heat), only one participant (3.3%) at the end of the four weeks of the study reported mild pain at the application site of the formulation with microparticles (F1), with rapid relief after a few minutes of application.

Regarding the clinical evaluation of skin hydration (opacity and roughness), no change was observed in D0 or D28. Data concerning the evaluation of the subjective parameter of hydration indicate that, at the application site of the formulation with microparticles, (F1) there was an improvement in the sensation of hydration reported by 37.1% of the participants on the second day and in all participants in the last day. In the left forearm, formulation without microparticles (F2), there was an improvement in the sensation of hydration in 48.6% on the second day and in all participants on the last day. The control was evaluated with better hydration by 2.9% of the participants on the second day and by 96.7% on the last day.

## Discussion

As to age, the participants in this study had an average of 26.3 years (Standard deviation 7.80; minimum 19; maximum 59). The variability in the age of study participants may predict the action of the product in different stages of skin aging. This variation in the participants’ age has been observed in other studies that evaluated the safety and efficacy of new formulations^36^ ^-^ ^38^.

Concerning sex, 30 (85.7%) of the participants were female (Table 1). Skin characteristics such as erythema, melanin, elasticity, thickness, transepidermal water loss and pH vary in the different anatomical sites between men and women and also in the different age groups^39^ ^-^ ^42^. Authors point out the importance of considering variations in the biophysical properties of the skin at different ages, genders and anatomical locations because these differences are related to individual susceptibility to skin diseases; they should be considered in studies and in the production of skin products^41^.

The same occurs with BMI. This variable influences skin quality and, therefore, selecting a sample with a wide variation of BMI is important to understand the action of the product in a broader way. In this study, BMI also varied considerably, with a mean of 23.2 (Standard deviation 4.42; minimum 16.1; maximum 32.1).

The quantification of erythema showed that the tested formulation did not produce a local inflammatory response, since there was no difference between average erythema in D0 and D28 (p = 0.8650). The first event after the onset of the inflammatory response is vasodilation with increased local blood flow, followed by increased vascular permeability. These phenomena are promoted by chemical mediators and are clinically translated into the onset of erythema^43^.

The evaluation of melanin showed a difference between the averages of the evaluated sites in both D0 (p = 0.0037) and D28 (p = 0.0053). These findings demonstrate that the amount of melanin was not homogeneous at the four sites evaluated at D0 and D28. Melanin levels may vary in the different parts of the body^41^. Besides anatomical issues, this fact can be explained by difference in sun exposure^41^, corroborating with the findings of this study. Personal factors such as age, sex, race, anatomical site and skin surface properties, as well as environmental factors such as light conditions, temperature, humidity and climatic variations can influence the color of the skin^41^ ^,^ ^44^.

The function of melanin is the protection of the DNA of the keratinocytes against radiation^45^; it is known that its concentration, its type and its location represent important factors in the evaluation of skin color, as well as in the evaluation of blood flow, thickness, softness and degradation of skin proteins^46^. This fact represents an important measure in the evaluation of possible skin changes.

The evaluation of the amount of melanin showed a decrease in the average values only at the application site of the formulation with microparticles (Figure 3), although the analysis did not indicate a statistically significant difference (p = 0.8592). This fact may indicate a possible photoprotective effect of the formulation with microparticles, since this reduction was observed only at the application site of this formulation (F1).

A study that evaluated the effects of a tamarind-containing emulsion on melanin identified a reduction in the amount of melanin at the application sites and attributed this result to the presence of phenolic compounds present in the extract^47^. It is known that chamomile contains several phenolic compounds in its composition, a fact that suggests the need for future studies to better investigate this property.

In the pH evaluation, there was no difference between the sites in D0 (p = 0.0819), but there was a significant difference in D28 (p = 0.0019). Furthermore, the mean pH of the skin was similar in the four sites evaluated at D0, but there was a change between values in D28, without, however, changing the physiological values. As this change was also verified in the control of the same arm, it can be inferred that this increase is not related to the use of the formulation. Furthermore, this alteration did not interfere in the values of pH normality in the skin.

pH is also a variable that varies in the different body regions^42^. In this study, it is believed that the difference is the result of the application of the formulation with chamomile, which caused an increase in its mean value, despite remaining within the physiological limits.

No significant alterations were found in the subjective evaluations of burning, pruritus and heat, or in the clinical evaluation of desquamation. It is known that the three components used are released by regulatory agencies, studied in different types of research and used for various purposes, among them skin treatments and care.

Chamomile extract was evaluated for toxicity, presenting safety at the dosages recommended for humans, without cytotoxic, genotoxic or mutagenic effects^48^. The use of chitosan in nanocapsules with alginate, for the treatment of infectious or inflammatory conditions of the skin showed antibacterial, anti-inflammatory and controlled release activity, without causing skin irritation^49^. A study on the toxicity of lanolin and its effect on animal cicatrization concluded that it has no toxic effect on monocytes, important cells of the cicatrization process^50^.

As for the participant who was removed from the study in D1 due to a report of local reaction, the event is attributed to a possible unknown personal sensitivity to the components of the formulation, since this was an isolated case with spontaneous regression of signs. The participant who reported a sensation of pain indicated that the pain had a mild degree, with short duration, and it was spontaneously relieved.

The evaluation of the skin barrier function showed a significant increase in the average of transepidermal water loss at the four evaluated sites (pF1 = 0.0003, pC1 < 0.0001, pF2 = 0.0004 and pC2 < 0.0001), indicating a decrease of the function (Figure 3). Variations in transepidermal water loss can be attributed to blood flow, skin temperature, lipid content of the stratum corneum and degree of corneocyte formation^41^ ^-^ ^42^. The increase in transepidermal water loss through the stratum corneum in the present study can be attributed to the period of the year and to the dry climate of the city in which the study was conducted. Studies maintaining a longer evaluation time may elucidate the possible influence of these aspects.

The comparison of the results of the four sites evaluated (Table 3) showed that there was no difference in the average hydration values at D0 (p = 0.4837) and D28 (p = 0.1348). As for the average values of transepidermal water loss, a difference was observed between the groups at D0 (p = 0.0006) and D28 (p = 0.0029). This data demonstrates non-homogeneity in the amount of transepidermal water loss between sites assessed at the beginning and at the end of the study.

The hydration analysis at the end of the study evidenced an increase in the average of this coefficient in all the evaluated sites; only the site where the formulation with microparticles was applied (F1) (p = 0.0483) and the right control (C1) ( p = 0.0413) presented a significant difference (Figure 3).

Currently, the prevention of skin lesions is performed according to the type of injury. Products of various brands are available for application to the whole skin aiming at the formation of a protective film and, thus, promoting protection against physical or chemical aggressors. However, these products do not present chitosan microparticles with chamomile; microparticles that promote an increase in the stability of the encapsulated botanical extract and also the slow and controlled release of their actives that can be a differential.

The study had as possible limitations the non-randomization of the application sites and the non-measurement of the exact amount of the product to be applied.

It is, therefore, a technological product that uses in its composition an active principle with proven biological activities with the advantage of presenting a controlled release, added in a base of known use and easy incorporation. Furthermore, these results stimulate the continuity of the use of this formulation in animal tests for the evaluation of its action in the cicatrization of lesions and future clinical studies to evaluate its effect in the prevention and also in the treatment of skin lesions such as radiodermatitis, peristomal lesions and pressure lesions, which are areas of interest for nursing.

## Conclusion

The hypothesis that the lanolin formulation, containing *Chamomilla recutita* (L.) rauschert microparticles coated with chitosan would be safe for application to the intact skin of healthy volunteers was confirmed. Despite an isolated case of erythema, this event was not observed in other participants. There was alteration in melanin, attributed as protective effect of the formulation. There was no desquamation; one participant reported pain, with spontaneous and brief regression. The formulation did not cause pruritus or burning in the analyzed period.

The results showed an improvement in skin hydration at the application site of the formulation with chamomile; the subjective evaluation of the sensation of hydration by the volunteers at the end of the period was positive in all evaluated sites.
